# A Continuum Model of Actin Waves in *Dictyostelium discoideum*

**DOI:** 10.1371/journal.pone.0064272

**Published:** 2013-05-31

**Authors:** Varunyu Khamviwath, Jifeng Hu, Hans G. Othmer

**Affiliations:** 1 School of Mathematics, University of Minnesota, Minneapolis, Minnesota, United States of America; 2 Digital Technology Center, University of Minnesota, Minneapolis, Minnesota, United States of America; Karolinska Institutet, Sweden

## Abstract

Actin waves are complex dynamical patterns of the dendritic network of filamentous actin in eukaryotes. We developed a model of actin waves in PTEN-deficient *Dictyostelium discoideum* by deriving an approximation of the dynamics of discrete actin filaments and combining it with a signaling pathway that controls filament branching. This signaling pathway, together with the actin network, contains a positive feedback loop that drives the actin waves. Our model predicts the structure, composition, and dynamics of waves that are consistent with existing experimental evidence, as well as the biochemical dependence on various protein partners. Simulation suggests that actin waves are initiated when local actin network activity, caused by an independent process, exceeds a certain threshold. Moreover, diffusion of proteins that form a positive feedback loop with the actin network alone is sufficient for propagation of actin waves at the observed speed of 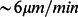. Decay of the wave back can be caused by scarcity of network components, and the shape of actin waves is highly dependent on the filament disassembly rate. The model allows retraction of actin waves and captures formation of new wave fronts in broken waves. Our results demonstrate that a delicate balance between a positive feedback, filament disassembly, and local availability of network components is essential for the complex dynamics of actin waves.

## Introduction

Active cell movement is critical at various stages in the life cycle of most multicellular organisms, and movement entails force generation within cells and mechanical interactions with their surroundings. The mechanical interactions are mediated by the cytoskeleton, which is a network of actin filaments, intermediate filaments, and microtubules in the cytoplasm. Experimental studies have shown how actin polymerization and actomyosin contraction lead to force generation within a cell, and have led to detailed maps of actin flow patterns within certain moving cells. They reveal large regional variations within a cell in the actin network density, and the levels of myosin, nucleation factors, filament binding proteins, and other control species that modulate network properties. It is widely-appreciated that dynamic spatial and temporal control of network properties is essential for cell motility, but the complexity of both the signaling networks and the cytoskeletal network has hindered progress toward an integrated model.

Cytoskeletal reorganization, particularly of filamentous actin (F-actin), is an essential component of motility in eukaryotic cells. They are able to migrate in the absence of external chemoattractants by continuously rebuilding the cytoskeleton and periodically extending pseudopods at random membrane locations. In the absence of directional signals neutrophils and *Dictyostelium discoideum* (Dd) explore their environment randomly [1], [2], and thus the intracellular networks that control the mechanics must be tuned to produce signals that generate this random movement. In neutrophils three Rho GTPases Cdc42, Rac and RhoA, which are activated by Ras, control three pathways that lead to the assembly of filopodia [3], the formation of lamellipodia [4], [5], and the contraction of the F-actin networks, respectively. In mammalian cells activation of RhoA leads to inactivation of MLCPase, an inhibitor of myosin contraction [6], and thereby to contraction. Rac activates factors such as ezrin, which localizes at points of actin fiber attachment, and facilitates nucleation of actin polymerization by regulating Arp2/3 [7]. The balance between the RhoA and Rac pathways determines whether dendritic network formation or bundling of F-actin dominates in neutrophils, while in Dd the balance is between the Rac and cGMP pathways [8]. Details of the pathways involved are discussed later.

In the presence of a chemotactic signal the cells must orient properly, which means the dynamical system controlling the mechanics must respond to the bias. It is known that phosphatase and tensin homolog (PTEN), which converts phosphatidylinositol-3,4,5-trisphosphate (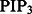) to phosphatidylinositol 4,5-bisphosphate (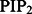), is a major regulator of migration during chemotaxis in both Dd and neutrophils [9], [10]. Activated PI3 kinase (PI3K), which phosphorylates 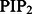 into 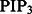, is increased at the site of signal reception and PTEN localizes at the lateral and posterior regions of migrating cells. This leads to a reduction of 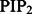 at the leading edge, which may enhance competition between blebbing induced by membrane detachment from the cortex and actin-driven pseudopod formation by localization of 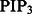 production there. Myosin II, and hence contraction, is localized at the posterior end of migrating neutrophils and Dd [11]. Whether PTEN controls myosin II localization is not known, but it is known that PTEN localizes at the side and rear prior to myosin II localization [12]. This suggests that PTEN may be involved in a positive feedback loop in which contraction enhances accumulation of PTEN and myosin II [12]. However, PTEN is not the sole controller of myosin localization, for it still localizes in *pten
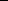
* cells, and this may involve the cGMP pathway in Dd or the RhoA/Rock pathway in neutrophils [13].

Integration of the mechanics with the intracellular networks that control actin, myosin and the other components involved in the mechanics is complex. Significant progress on understanding the early steps in the cAMP/G-protein coupled signal transduction network in Dd, in particular for adaptation and amplification of directional signals, has been made and will be described elsewhere [14]. The output of this module is activated Ras, another GTP-binding molecular switch, and PI3K, which is activated by Ras and produces 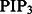. Here we focus on the downstream steps involved in the interaction of this signaling pathway with actin network formation, and in particular, study the effect of feedback from actin network formation to the Ras/PI3K component of the signaling network.

In the absence of chemotactic signals, different motile structures of F-actin consisting of foci and bands are observed on the substrate-attached cell surface in Dd [1], [15]–[19]. The motile bands propagate on the cell-substrate surface and their contact with the cell border can lead to membrane extension. It is also observed that the actin waves convert into phagocytic cups upon contact with external particles [20]. Treatment of Dd with latrunculin A (LatA), which sequesters globular actin (G-actin) and leads to dissolution of F-actin and the cortex, highlights both motile structures, as they become prominent during cell recovery from the drug treatment. These waves arise at the boundary between domains of high and low 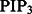 levels after treatment with latrunculin. The waves are often closed and of varying shape, and they propagate by treadmilling, as shown by actin recovery after photobleaching [21], [22]. Myosin-IB (MyoB), which links the actin network to the membrane [23], is found at the front of a wave, the Arp2/3 complex and a dense dendritic network are found throughout the wave, and coronin, which inhibits filament nucleation and indirectly regulates cofilin activity via dephosphorylation [20], [24], is found at the rear. Cortexillin, which organizes actin filaments into anti-parallel bundles, is found where 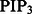 is low [25].

**Figure 1:**
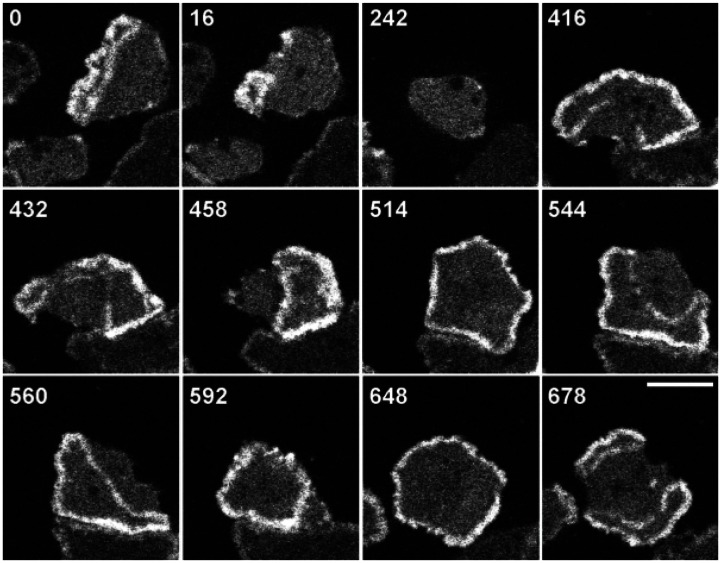
Actin waves in normal and LatA-treated cells. Evolution of actin waves in a LatA-treated cell imaged by TIRF displays expansion, retraction, separation, and collapse of the waves. The cell comes in contact with another cell between 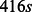 and 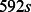 [25].

The waves are three-dimensional structures approximately 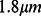 in width, with a steep front that extends 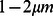 into the cytosol and gradually decaying back. Molecular components involved in actin waves have been identified using confocal microscopy and total internal reflection fluorescence microscopy (TIRF), which targets labeled species within a thin region near the cell-substrate interface (usually less than 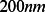). As depicted in Figure 1, actin waves are generally formed as a closed band which continuously alters its shape by propagating, expanding, retracting and abruptly changing direction. A moderate level of F-actin is found within the enclosed area, connecting wave fronts [21], [26]. Wave fronts are capable of abruptly changing their direction of propagation, causing an expanding enclosed area to retract and a retracting area to expand. A closed actin wave can break into spiral waves which later form separated closed waves. Broken waves and newly connected waves may also shrink and collapse. When wave fronts enclosing two separate regions collide, both wave fronts are annihilated and the enclosed regions fuse [15], [21], [25], [26]. The propagation speed of the wave fronts is around 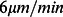.

Fitzhugh-Nagumo equations, describing F-actin density, various partners, and sometimes average filament orientation, have been used to model formation and motility of actin foci and patches [27]–[29]. These models demonstrate that actin foci may result from random fluctuations in filament nucleation, and that the foci can become motile and develop into wave fronts when filament orientation is incorporated. Others have proposed models which couple individual-filament dynamics and various schemes for regulation of nucleation promoting factors (NPFs), including FitzHugh-Nagumo type activation [30] and active NPF transport via F-actin [31], [32]. For suitable parameters, the uniform state in these models may be unstable and admit periodic solutions corresponding to either motile spots or traveling waves, depending on parameter values [30]–[32]. In addition to the Fitzhugh-Nagumo type models, an activator-inactivator model for actin waves has been proposed by Carlsson [33]. In this model, spontaneously-activated NPF induces and is subsequently inhibited by autocatalytic F-actin network formation. Detailed filament dynamics, including branching, barbed-end capping, filament severing, and filament orientation, are incorporated. Stochastic simulations display motile spots, traveling waves, and their network structure. The waves generated by the model annihilate when they collide, which is consistent with observations.

Although the existing models are able to reproduce motile spots and traveling waves, they generally fail to capture all aspects of the experimentally-observed network structure, characteristics, and dynamics of the propagating actin waves. The waves produced by these models are propagating open fronts in contrast to closed actin waves generally observed in experiments. Most models represent the actin network by a single variable and cannot predict the detailed network structure. Although stochastic simulations of the activator-inactivator model display the network structure and correctly predict the behavior of colliding waves, they do not capture the back of actin waves, continuously propagating fronts, and more complicated behaviors. Moreover, details of the interaction between the molecular constituents involved in the waves have been largely neglected in the existing models. Typically initiation of the actin waves is caused by fluctuations of the actin density or spontaneous activity of NPF, while wave propagation is due to polymerization or diffusion of F-actin itself. Most importantly, retraction of actin waves, which partly contributes to the complex behaviors of the waves, is not captured by existing models. In particular, the FitzHugh-Nagumo model, on which many models are based, does not allow reactivation in the decaying region of actin waves, thereby rendering these models incompatible with retracting actin waves.

**Figure 2:**
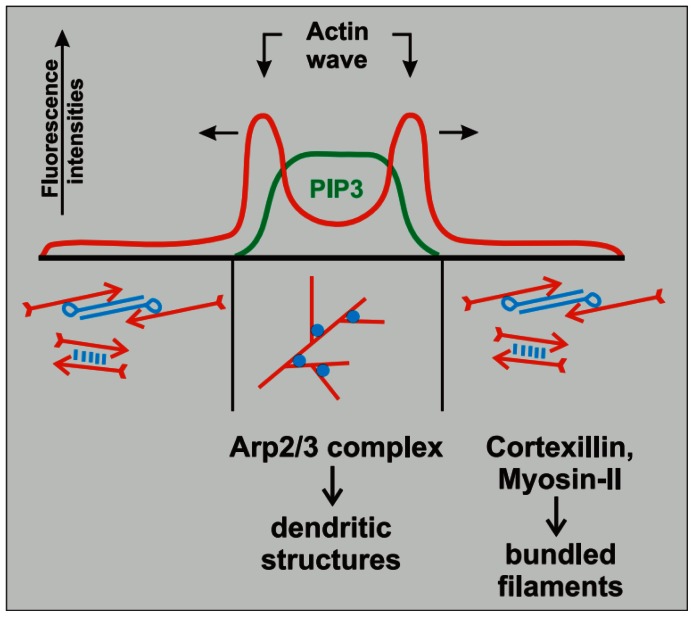
The spatial localization of components in an actin wave network, modified from [22]. The diagram represents F-actin and 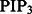 densities along a TIRF line scan through a closed actin wave in a cell attached on a glass surface. The wave fronts are propagating outwards while 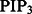 localization coincides with the dendritic network comprising the actin wave.

We propose a model of actin wave initiation and propagation that accounts for both filament dynamics and interaction with the PI3K signaling pathway, which is absent in existing models. In particular, the activity of this pathway is necessary for actin waves and is represented by 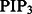 localization, which is specific to the area enclosed by the actin waves as shown in Figure 2. Our model, which incorporates a simplified PI3K pathway, includes a continuous description of actin network with similar details to the discrete description in [33]. To develop a computationally-feasible model, we assume that filaments are aligned normal to the membrane and omit filament capping and severing. The assumption on filament orientation does not permit lateral polarization of actin filaments and enables us to show that network propagation is also possible via diffusion of a cytosolic protein in the PI3K pathway. In future work, when membrane contraction induced by actomyosin activity is incorporated, this assumption will be relaxed. Other implications of the assumptions underlying the model will be discussed in detail later. Our model is used to capture characteristics and behaviors of propagating actin waves in PTEN-deficient cells – which are similar to waves in normal cells, except that they do not retract [34]. In the PTEN-deficient cells, when an actin wave is initiated, the enclosed area, which may split, continues expansion until it collapses. When surrogate PTEN activity is imposed in our model, abrupt changes in propagation direction of actin waves into retracting waves and formation of new wave fronts are observed as in wild-type cells. Moreover, the model exhibits correct localization and dynamics of associated molecules. In particular, it is able to explain localization of 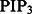 in the area enclosed by actin waves. Finally, our model suggests that actin waves can be initiated by actin precursors, or spots, which are driven by other processes such as membrane adhesion and internalization of clathrin-coated structures [19], [25]. Before developing our model, we will discuss the underlying biochemistry in more detail.

### Biochemistry of Actin Waves

Experimental observations indicate that Arp2/3, an actin branching nucleator, is enriched throughout actin waves, suggesting waves are composed of dendritic actin network. In addition to Arp2/3, certain molecules including MyoB, CARMIL, and coronin are selectively associated with propagating fronts of the actin waves. CARMIL is an adaptor protein that binds to MyoB, Arp2/3, and capping protein *in vitro*. The roles of MyoB and CARMIL are unclear although CARMIL binding to capping protein has been linked with inhibition of barb-end capping. Coronin is known to destabilize and dissociate actin branches [24]. It can also cooperate with cofilin and actin-interacting protein 1 (Aip1) to abruptly disassemble actin filaments *in vitro* [35], [36].

**Figure 3:**
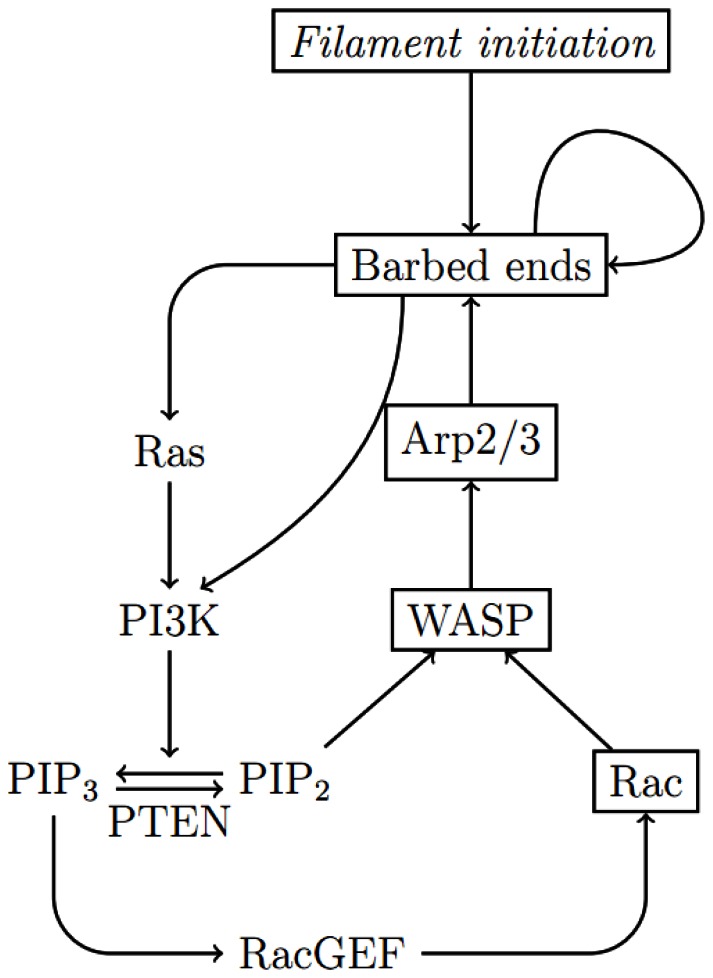
A simplified diagram for the feedback between F-actin and PI3K. This diagram shows regulation of molecular activity related to branching of actin filaments. Arrows represent activation, except for the conversion between 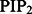 and 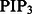. The actin wave model developed herein describes the dynamics of boxed components, and the condensed network captures the essential features of wave propagation.


The branching of actin filaments via Arp2/3 occurs near the plasma membrane [37]. This nucleation activity is part of a regulatory network containing positive feedback, as depicted in Figure 3. The branching activity of Arp2/3 is facilitated by class I nucleation promoting factors (NPFs) such as SCAR/WAVE and WASP. These NPFs localize to the plasma membrane by binding to phospholipids, and form a complex with Arp2/3 and G-actin that facilitates their binding to existing filaments to create new branches. Activation of the NPFs and creation of the dendritic actin network in *Dictyostelium* occurs downstream of Rac, a small GTPase regulated by 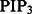 activity [38]–[40]. WASP is normally associated with clathrin-coated pits and can assume the role of SCAR/WAVE at pseudopods in SCAR/WAVE-null cells. As actin waves also exist in these cells, WASP regulated branching alone is sufficient [21], [41].

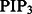
, which is produced via phosphorylation of 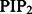 by PI3K, serves as a membrane docking site for activation of RacGEFs, which are guanine nucleotide exchange factors of Rac. Activated RacGEF in turn facilitates Rac exchange of GDP for GTP, converting Rac into the active state. F-actin nucleation activity via Rac is dependent on local 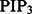 density. Moreover, the activity of 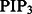 is essential for formation of the actin waves. Disruption of PI3K activity by LY294002 completely abolishes active actin waves on the cell-substrate surface within 2 minutes and the actin waves recover within 3 minutes after removal of the drug [20], [22], [34]. Unlike Arp2/3, 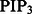 is enriched in the area enclosed by actin waves, with a transition zone at the wave fronts and low 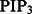 density outside the waves. 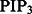 can be dephosphorylated into 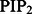 by PTEN, which is generally found within the external area, along with Cortexillin I, an actin-filament bundling protein, and myosin II. Three-dimensional images show that PTEN is not only enriched in the external area of the cell-substrate surface, but also throughout the nonattached area of the cell membrane. Because 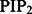 serves as a specific binding site for PTEN membrane localization, the observed PTEN membrane-localization profile may be due to local depletion of 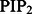, which is otherwise abundant, within the region enclosed by actin waves. PTEN activity is not necessary for the existence of actin waves, as they are observed in PTEN-deficient cells. However, actin waves in these cells continuously expand without retraction, suggesting a role for PTEN in reversal of the direction of propagation of the waves [25], [34].

The feedback from F-actin to PI3K and Ras, a PI3K activator whose activity colocalizes with 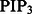 in actin waves, closes a positive feedback loop between F-actin, NPFs, and 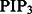. This F-actin dependent activity of Ras and PI3K has been observed in pseudopodia of *Dictyostelium* cells [42]. We hypothesize that the feedback loop coupled with autocatalytic nucleation activity of existing filament barbed ends due to branching can serve as a basis for the spontaneous and sustained activity of actin waves, and we develop our model using the minimal necessary components of the feedback loop including Rac, WASP, and Arp2/3, that preserve this network structure. A more complete description of the signaling pathway may be found in the literature [8], [43], [44].

In addition to the 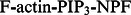 cascade, actin waves are regulated by signaling from other intracellular structures. Actin waves can be initiated at clathrin-coated pits as WASP is recruited to assemble branched actin network during endocytosis [25], [41]. Because actin waves are observed predominantly at the substrate-attached surface, origination of actin waves without the clathrin-coated pits is potentially linked to substrate-adhesion. Interestingly, actin structures resembling actin waves are observed in phagocytic cups during particle uptake [20]. Moreover, integrin is observed along with adhesive-actin waves in fibroblasts, although these waves are characteristically different from the actin waves in *Dictyostelium*. Frequency of the fibroblast actin waves is greatly reduced when integrin activity is inhibited or cells are placed on poly-L-lysine-coated coverslips. In higher eukaryotes, the dependence on substrate adhesion may arise via the integrin-PI3K-Rac signaling cascade [19]. While *Dictyostelium* does not have integrins, it has an integrin homolog, SibA [45]. Because actin waves are found in myosin-II-null cells, they are independent of actomyosin network activity and chemical stimulation.

## Results

### The Actin Wave Model

We begin by developing a mathematical model to explain the primary characteristics of growth and propagation of the actin waves based on the known molecular interactions. The complete model is comprised of a continuum component for the F-actin network dynamics and a simplified description of the PI3K pathway. The formulation of the former is done in two steps, as described in the Analysis section. There we first we formulate a discrete model based on actin monomers and then reduce it to a continuum description. Here we describe the biochemical basis of the model and then present the equations that govern the integrated actin and PI3K networks.

Because the F-actin structures associated with actin waves are restricted to cell regions close to the cortex, especially at the cell-substrate interface, we assume that actin filaments only grow from the substrate-attached membrane of a cell placed on a flat surface. To reduce the computational complexity of the model, we assume that filaments are always attached to the cell membrane at the barbed end, and that filaments within the structure are oriented vertically and tethered to the cell-substrate interface. Thus we neglect the fact that side branches are generally not parallel to the parent branch, but this is not a critical factor since we do not incorporate mechanical forces in the network. Furthermore, the foregoing implies that diffusion of actin filaments is neglected, as is filament severing by cofilin and coronin.

**Figure 4:**
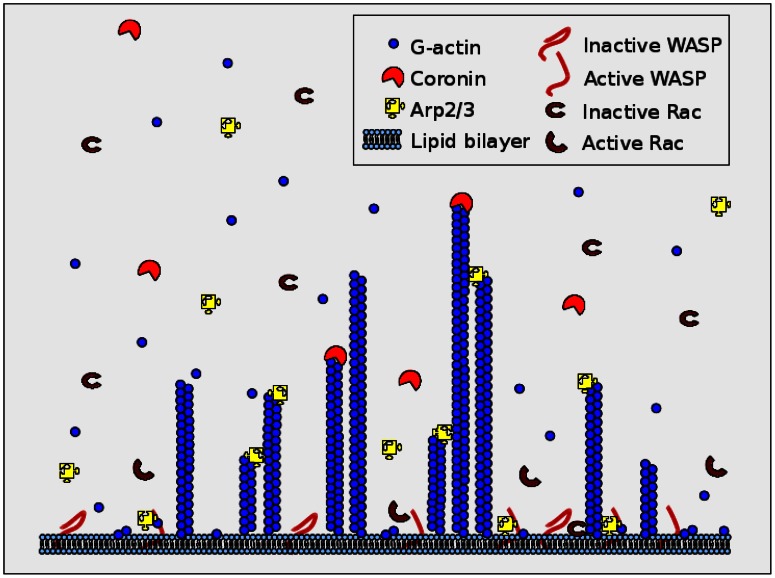
A schematic of the network structure and molecular interactions in the model.

The dendritic network is a collection of F-actin filaments that polymerize at a rate proportional to the local G-actin density and depolymerize at a fixed rate. As shown in Figure 4, the network consists of three types of actin filaments according to the state of their pointed ends: filaments with free pointed ends (whose local concentration is denoted by 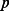), new branches with pointed ends protected by Arp2/3 (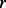), and destabilized branches with coronin at pointed ends (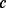). actin waves is associated with the substrate-attacted membrane, we assume that it only grows at the surface, each filament growing at a rate determined by the local concentration of the membrane-bound G-actin. For simplicity, we assume that polymerization and depolymerization of filaments only occur at barbed ends and pointed ends respectively. Moreover, since filaments are tethered and aligned normal to the flat membrane, there is no lateral polymerization of filaments, and therefore they can be represented by their pointed-end density. New filaments are nucleated at the membrane either by dimerization of G-actin or by branching from an existing filament, the latter of which is facilitated by a branching complex of WASP, Arp2/3, and G-actin. On the other hand, detachment of actin branches is regulated by coronin, and involves two steps. First, coronin binds to a filament pointed end, replacing Arp2/3, and then bound coronin spontaneously detaches the branch and is released, leaving a free pointed end that can be depolymerized [24]. Filament capping and severing are omitted from the model because they lead to filaments with detached barbed ends, which will increase the dimension of the simulation domain. We have also developed a model which includes barbed-end capping (not shown here), but simulations of this model are prohibitively time-consuming, and simplifications are needed. *In vivo*, these activities will limit growth of barbed-end density and facilitate decay of the back of actin waves. Because we cannot track individual filament connections, the possibility that branches are broken by depolymerization of mother filaments is omitted. Thus there are two mechanisms for filament disassembly in the model – by depolymerization of dimers, and by debranching of branches with only one G-actin subunit. We discuss the effects of omitted disassembly mechanisms, including filament capping and severing, on the dynamics of actin waves later.

In the Analysis section we describe the detailed discrete monomer-based model for the F-actin network and show how to obtain the corresponding continuum model used in the simulations. As there is no lateral interaction within the F-actin network, other than by competition for diffusible species, we first develop a discrete network description in one spatial dimension along the filament length, assuming that the horizontal composition of all mobile species is uniform. Then approximations are made to obtain a continuous description. Finally, diffusion of free molecules is introduced in the two-dimensional continuous models. Note that in these descriptions, we have not introduced membrane binding of G-actin, and in effect assume rapid equilibrium between membrane-bound and free G-actin. Dimerization, branching, and polymerization are assumed to be dependent on the amount of free G-actin at the barbed ends. Since all variables are functions of time, omission of 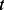 from the variables, except when it is explicitly specified, is assumed to simplify the notations.

In addition to the F-actin network, the positive feedback through the PI3K pathway, which promotes filament branching via activation of Arp2/3, is an essential component of actin waves. In this paper we model actin waves in PTEN-deficient cells, and therefore PTEN dynamics are not incorporated. We then simplify the pathway, as depicted by boxed components in Figure 3, so that a minimal number of the intermediate effectors are included. The simplified network involves Rac (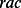), WASP (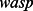), and Arp2/3 (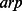), in both activate and inactive forms, as well as the complex formation that leads to nucleation of actin branches. The reactions in which they participate are as follows.
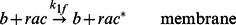


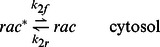


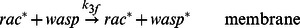


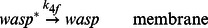


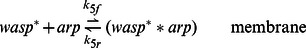


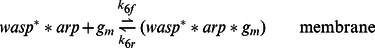


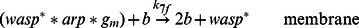



Here 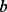 denotes the local density of all barbed ends. In reality, RacGEF diffuses within the cytosol and its activation occurs at the membrane and is regulated by F-actin activity via 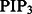, but it is not known if Rac activation by RacGEF is restricted to the membrane. In our model we combine these activation steps, as well as the upstream PI3K activity, and assume that Rac is diffusible and has a low spontaneous activation rate in the cytosol, whereas the primary activation occurs via interaction with actin filaments at the membrane. Because WASP binds to both 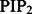 and 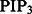 [38], we assume a constant WASP density at the membrane, which implies that WASP activity at the substrate-attached surface is regulated solely by activated Rac activity while its localization is relatively constant. Activated WASP then recruits Arp2/3 to the membrane, forms a complex with G-actin, and nucleates a new branch on an existing filament. We assume minimal membrane diffusion. A similar result with membrane-bound species diffusing at 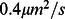 and higher Arp2/3 cytosolic density will be shown as well.

The dynamics of cytosolic and membrane densities of molecular species are modeled by reaction-diffusion equations, while interactions between species are assumed to follow mass-action kinetics. The simplified description of the PI3K pathway is then combined with the continuum F-actin model given in the previous section, where filament nucleation and polymerization depend on the local density of membrane-bound G-actin. The resulting equations consist of cytosolic variables described on the domain 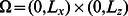 (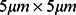 unless otherwise noted), and membrane variables defined on 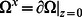. All variables that appear in the model are summarized in Table 1. The evolution of the cytosolic variables is described by the system
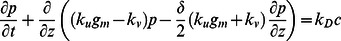


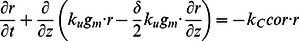


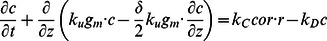


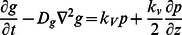


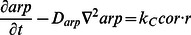


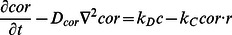


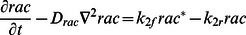


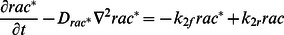



**Table 1:** Variables in the actin-wave model.

Molecular species	Description
Cytosolic	
*p*	Free pointed ends
*r*	Arp2/3-capped pointed ends
*c*	Coronin-capped pointed ends
*g*	Free G-actin
*arp*	Free Arp2/3
*cor*	Free coronin
*rac*	Inactive Rac
*rac* ^*^	Activated Rac

Membrane	
*b*	Barbed ends
*p*2	Pointed ends of dimers
*r*1	Arp2/3 branch of length 1
*r*2	Arp2/3 branch of length 2
*c*1	Coronin branch of length 1
*c*2	Coronin branch of length 2
*wasp*	Inactive WASP
*wasp* ^*^	Activated WASP
*wasp* ^*^*arp	WASP-Arp2/3 complex
*wasp* ^*^*arp*g_m_	WASP-Arp2/3-actin complex
*g*_m_	Membrane-bound G-actin

in 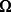 where 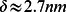 is the length of an actin subunit in F-actin. Refer to Table 2 for description and values of other parameters. All boundary conditions are specified by fluxes, which are

**Table 2:** Parameter values used in the actin-wave model.

Parameter	Value	Description	References
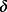	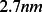	Actin-subunit length in filaments	[57]
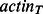	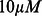	Total F-actin concentration	[33]
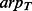	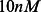	Total Arp2/3 concentration	
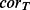	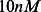	Total coronin concentration	
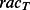	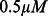	Total Rac concentration	
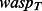	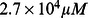	Total WASP density on the interface	
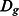	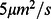	G-actin diffusion constant	[58]
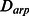	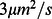	Arp2/3 diffusion constant	[59]
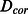	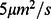	Coronin diffusion constant	
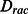	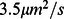	Rac diffusion constant	
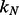	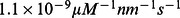	Dimerization rate constant	
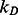	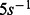	Branch-detachment rate constant	
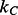	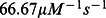	Coronin binding rate constant	
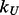	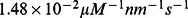	polymerization rate constant	
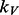	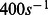	depolymerization rate constant	
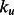	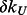	polymerization speed constant	
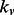	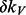	depolymerization speed	
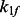	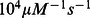	Rac activation by barb ends	
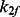	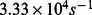	Spontaneous Rac deactivation	
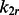	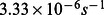	Spontaneous Rac activation	
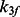	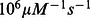	WASP activation by Rac	
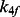	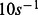	Spontaneous WASP deactivation	
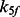	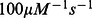	Arp2/3 binding to activated WASP	
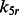	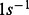	Spontaneous unbinding of Arp2/3	
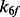	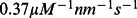	G-actin binding to WASP-Arp2/3	
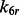	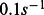	Spontaneous unbinding of G-actin	
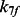	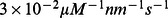	Branching	
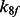	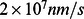	Membrane binding of G-actin	
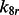	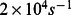	Membrane detachment of G-actin	



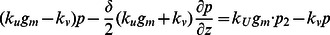


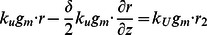


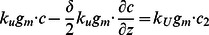


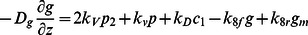


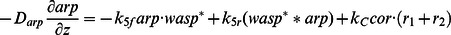


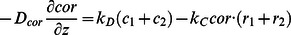


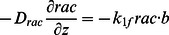


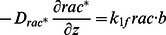



on 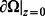. No-flux conditions are imposed on the other boundaries. The dynamics of short-filament membrane density – 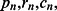 where 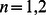 denotes filament length – is used to interface between continuous filament densities and filament nucleation at the contact surface 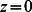. The evolution of the densities of these membrane-bound species, none of which diffuse on the membrane, is described by



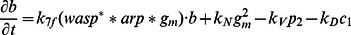


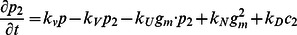


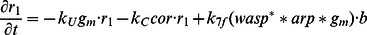


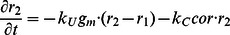


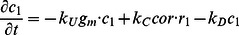


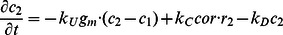


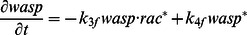


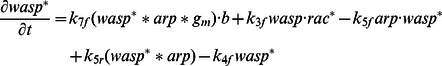


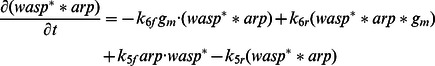


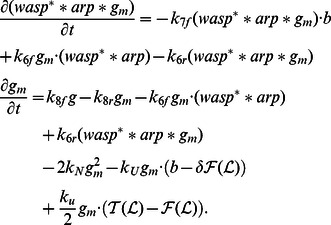



Here 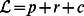 while 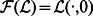 and 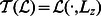 represent the pointed-end density at the lower and upper (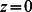 and 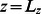), respectively. Parameters used for numerical simulations are presented in the Analysis section.

### Initialization and Characteristics of the Actin Waves

*In vivo* the actin waves may be initiated by various factors including clathrin-coated structures, SibA activity on the substrate attachment, or random fluctuations in filament dynamics. Each of these processes leads to local, and probably transient, accumulation of F-actin. While we consider this localization as a precursor for actin waves, we do not distinguish how a precursor is originated and model it generically by transiently increasing the actin dimerization rate in a narrow region on the lower surface of the cell.

**Figure 5:**
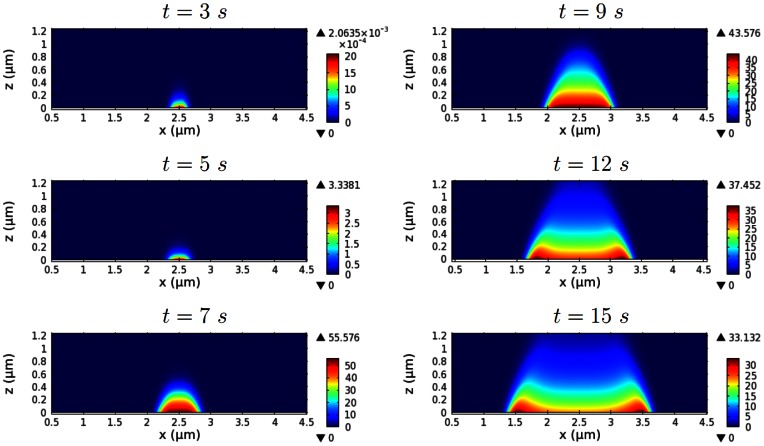
The development of actin waves from localized F-actin activity. The time course of the F-actin concentration (in 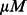) shows accumulation of F-actin at a spot, spot expansion, and separation of the wave fronts.

Figure 5 depicts early network growth and the separation of wave fronts. A transient increase in the dimerization rate constant (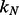) on a 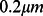 interval of the contact surface for a duration of 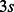, beginning at 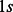, induces a burst of F-actin network growth in the interval. Initially the network grows primarily in the vertical direction and the local filament density near the membrane increases. Lateral propagation or expansion of the network is slow until the F-actin density reaches its peak at 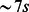 and the network reaches a certain height. Then the network rapidly extends both horizontally and vertically, becoming a large spot, before the center collapses and the network splits into wave fronts propagating outward in opposite directions, as shown schematically in Figure 2. The transition between the accumulation of F-actin intensity between 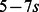 and the later expansion and separation of actin waves, can be explained as follows. First, during the initial growth phase, accumulation of F-actin is accelerated by the positive feedback via barbed ends at the contact surface. At the peak network density, local scarcity of Arp2/3 leads to reduced branching, which diminishes the positive feedback, and the peak F-actin density begins to decrease as filaments depolymerize. Finally, with further local depletion of Arp2/3, the dense region of F-actin at the initiation site of the wave decreases until a balance in filament and Arp2/3 turnover is reached, which explains the decay of the wave backs.

**Figure 6:**
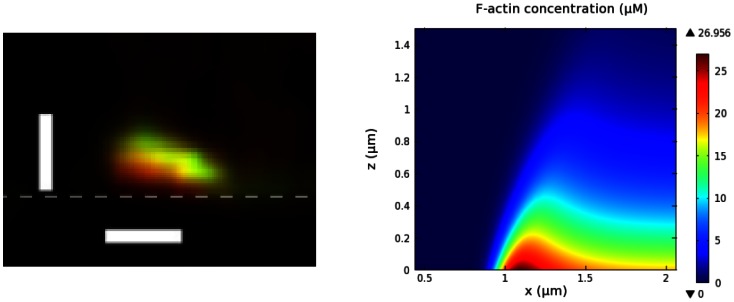
Shape of actin waves. *(Left)* A z-scan of an actin wave from [21], showing F-actin (red) and coronin (green). The dashed grey line approximates the bottom surface. Bars are 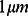. *(Right)* F-actin concentration within a simulated actin wave.

**Figure 7:**
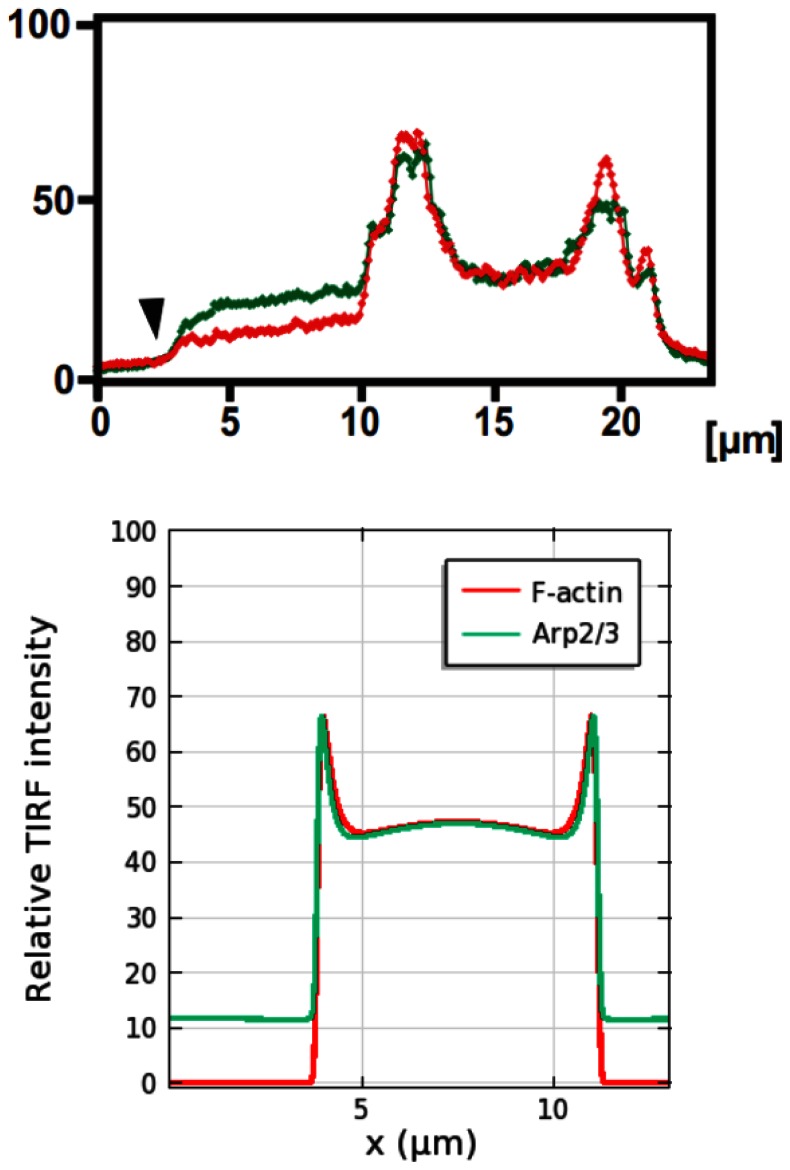
Localization of F-actin (red) and Arp2/3 (green) in actin waves. TIRF intensity along a line scan shows the relative localization of F-actin and Arp2/3 near the contact surface. *(Upper)* Experimental observation from [21]. *(Lower)* Simulation on a 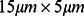 domain.

Thus the profile of the wave is high network density at the leading edges and a decreasing, but non-zero density, behind. A comparison of an experimentally-observed profile and a theoretically-predicted one is shown in Figure 6. One sees there that the typical actin wave profile obtained by simulation agrees well with a z-scan from a live cell [21]. The height of the simulated actin wave is 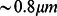 while the width of the wave front is 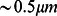, which compares favorably with the z-scan, when an intensity threshold at 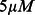 is applied to define the width. The wave fronts propagate at 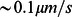, and both speed and intensity decrease slowly. The speed and intensity of the waves persist at the observed level in simulations on a larger domain (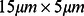), which suggests that the decrease on the smaller domain is due to limited availability of the molecular constituents. Arp2/3 density near the contact surface, as reflected in the computed TIRF intensity in Figure 7, closely reflects F-actin density, in good agreement with experimental observations [21]. The higher Arp2/3 density in the region outside the waves reflects the free Arp2/3 in the cytosol.

**Figure 8:**
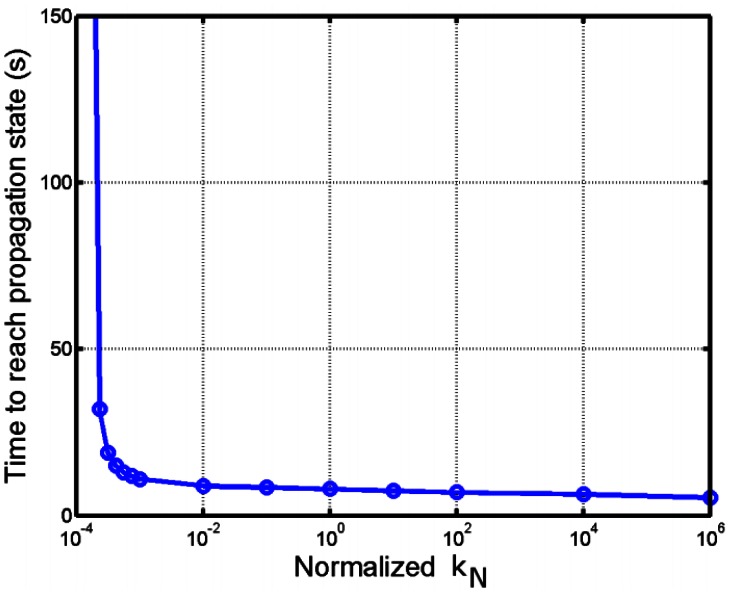
Dependence of actin-wave development on the precursor strength. Time required for actin waves to develop and propagate 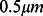 away from the nucleation center is plotted as a function of precursor activity. 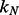 is normalized around the value used in other simulations.

As indicated earlier, different precursors may give rise to actin waves, and among them, clathrin-coated pits are the most easily observed. Because it is observed that not all clathrin-coated pits lead to actin waves, the initiation of actin waves may depend on accumulation of F-actin at sites of endocytosis before they disappear [25]. We studied this behavior by varying the activity level of actin wave precursors, proxied by the level of transient increase in the dimerization rate constant 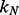 for a fixed duration of 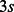. As depicted in Figure 8, actin waves do not form at low precursor activity. There is a threshold at which actin waves begin to form, and the initialization time rapidly decreases near the threshold. At higher stimulation levels the initialization time decreases slowly, approximately as a linear function of 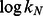.

Interestingly, the shape and speed of the actin waves do not depend on the precursor strength, but are rather dictated by rate constants and cytosolic levels of actin network components. Ten-fold changes in the initial nucleation strength, its duration, or its coverage affect neither height, speed, nor width of the propagating waves. We observe that the propagation speed is determined by a characteristic decay length of activated Rac and by the responsiveness of the positive feedback loop leading to branch nucleation. At a fixed propagation speed, the shape of the waves is determined by relative rates between various component processes. In particular, the inclination of the wave front is determined by the ratio between the propagation speed and the barbed-end polymerization rate, while the height of the waves is determined by the ratio between the polymerization rate and the branch-turnover rate. The half-width of the wave front, from its boundary to its vertical peak, is determined by the ratio between the propagation speed and the branch-turnover rate. Similarly, inclination and length at the back are controlled by the depolymerization rate.

### The Mechanism for Propagation of Separated Wave Fronts

Existing models suggest that propagation of actin waves is driven by F-actin diffusion or polymerization and branching of appropriately-oriented filaments, while collapse of wave backs follows the excitable medium paradigm. However, the F-actin network is highly interconnected, which suggests that the effective diffusion of the structure will be negligible. On the other hand, it is difficult to develop closed wave fronts with relatively constant propagation speed based on filament orientation alone. Moreover, the excitable media framework, based on Fitzhugh-Nagumo equations, does not allow retraction of wave fronts.

Because our model only allows vertically-oriented filaments, wave propagation is not driven by lateral polymerization of the network. Instead, it is diffusion of signaling molecules within the feedback loop, represented here by Rac, that drives wave propagation. Diffusion of locally-activated Rac leads to activation of nearby WASP, which in turn promotes branching of existing filaments and triggers the positive feedback loop and F-actin accumulation at the nearby sites. Moreover, simulation shows that this positive feedback is necessary for formation of actin waves, since dimerization, branching, and polymerization alone are not strong enough to organize the local actin network.

**Figure 9:**
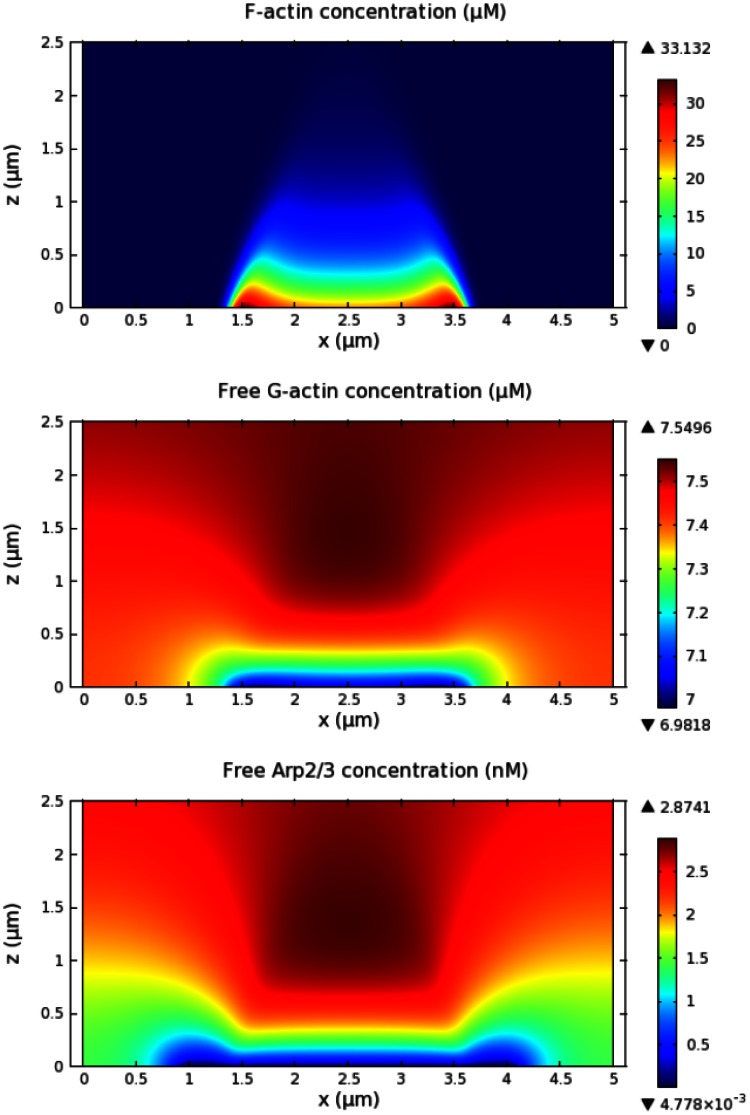
Scarcity of free Arp2/3 within actin waves. The distributions of free Arp2/3 *(bottom)* and G-actin *(middle)* are depicted with the corresponding F-acin density *(top)*. The Arp2/3 profile displays large spatial variation with low concentration near the contact region covered by the wave.

**Figure 10:**
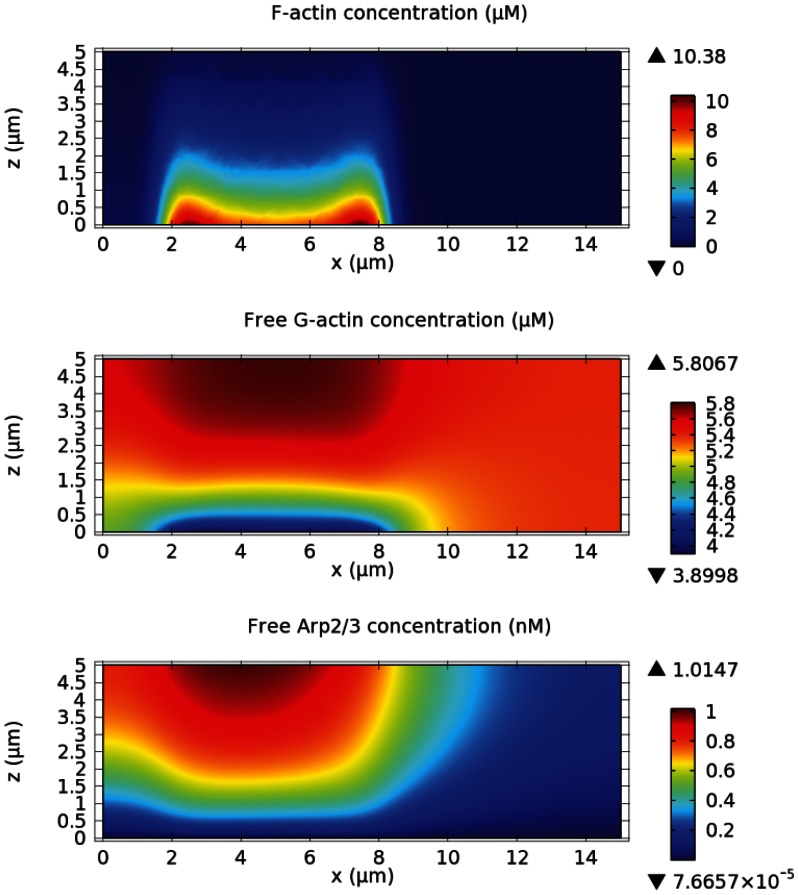
Actin waves at higher Arp2/3 concentration. The distributions of free Arp2/3 *(bottom)* and G-actin *(middle)* from a simulation on a 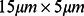 domain with 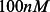 initial Arp2/3 concentration are depicted with the corresponding F-actin density *(top)*. Membrane diffusion of WASP and its complexes is incorporated in the simulation. 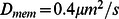, 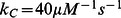, 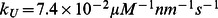, and 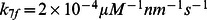.

On the other hand, we hypothesize that peaks in both height and density of the actin waves are determined by the scarcity of basic constituents of the network that are consumed behind the wave fronts as the waves expand. In this scenario, the suspension and the retraction of wave fronts are possible, and will be shown when PTEN activity is incorporated. In addition to the wave fronts, the mechanism based on scarcity leads to lower, yet active, F-actin activity in the region enclosed by the waves, which is also present in live cells. Fundamental components of the dendritic actin network include G-actin and Arp2/3, and either of them must be locally exhausted to create scarcity and overcome the autocatalytic branching due to positive feedback. As G-actin is much more abundant than Arp2/3, it is likely that majority of the scarcity effect is due to Arp2/3. In light of the absence of filament capping and severing in the model, we found that this happens when activation of an intermediate step in the positive feedback, *e.g.* WASP, is saturated. In addition, sufficiently high turnover of barbed ends, caused by dissociation of actin filaments, is required. In our model the spatial difference in free Arp2/3 and G-actin densities dominates the spatial variation in activated WASP, causing lower branching activity in the inner area where free Arp2/3 is diminished. Figure 9 shows that there is large spatial variation of free Arp2/3 and that free Arp2/3 density is very low in the area enclosed by actin waves. Although the Arp2/3 concentration (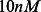) used in the simulations is in line with *in vitro* experiments [46], [47], it is relatively low. To preclude the possibility that decay of the wave back is sensitive to Arp2/3 concentration, we investigated if decay of the wave back occurs at higher Arp2/3 concentration. We increased Arp2/3 concentration to 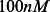 and included membrane diffusion of WASP and its complexes (at 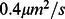, *cf.* [48]), and found that decay of the wave back still occurs. Figure 10 displays the density of F-actin, G-actin, and Arp2/3 in this numerical experiment. Analysis of the profiles shows that local depletion of G-actin is responsible for the decay of the wave back in this case. Note that some rate constants are adjusted to obtain a new balance between construction and destruction of the dendritic network. The propagation speed is significantly reduced to 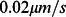 because of the slower branching rate, which is needed to suppress network growth outside the waves. *In vivo*, the suppresion of spontaneous network growth is more likely due to barbed end capping and filament disintegration by concerted cofilin, coronin, and Aip1 activities, which would lead to higher wave speeds at high Arp2/3 levels.

**Figure 11:**
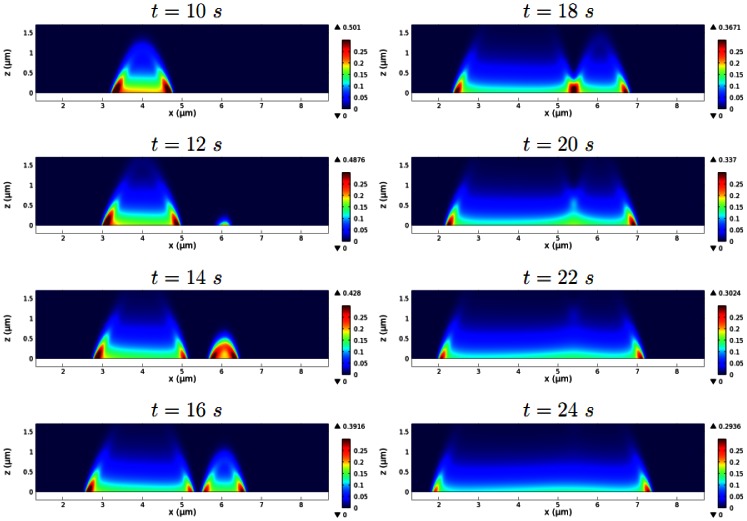
Annihilation of actin waves. Two wave fronts annihilate when they collide, combining the regions they enclose. The actin waves are initiated 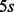 apart at 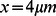 and 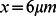 on a 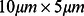 domain. The image sequence displays evolution of the pointed end density.

When two fronts of actin waves collide *in vivo*, both are annihilated, and the same is observed in simulations of the model, even when the wave fronts are not equally mature. An example of this is shown in Figure 11. Similarly, a wave front is annihilated when it reaches a boundary of the rigid simulation domain, displaying transiently-increased height when it is adjacent to the boundary.

### Localization of Coronin and 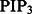


**Figure 12:**
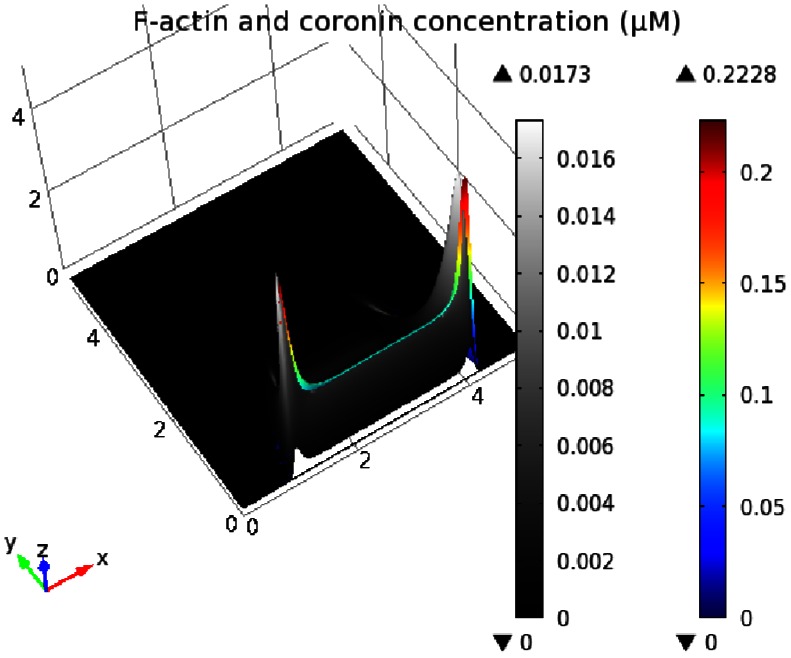
Relative coronin localization on the F-actin network in actin waves. Concentration of F-actin *(right bar)* and coronin-bound pointed ends *(left bar)* is plotted together, showing relative localization on the actin waves. The height displays relative concentration levels.

**Figure 13:**
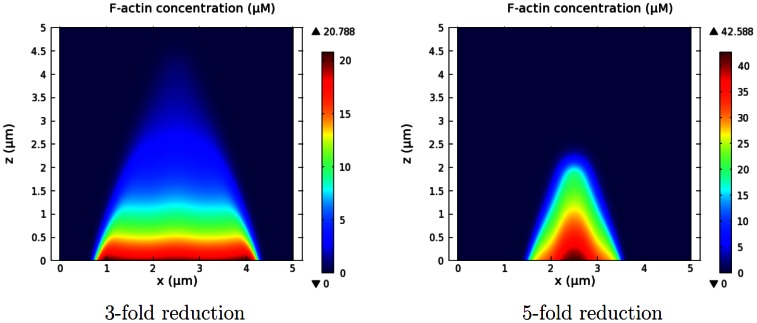
F-actin structures without coronin activity. Lower effective debranching rates caused by lack of coronin activity lead to altered actin structures. *(Left)* 3-fold reduction. *(Right)* 5-fold reduction.

Dissociation of barbed ends depends partly on debranching of actin filaments. In our model, coronin, which is observed at the top of actin waves in live cells, is responsible for debranching, but not disassembly, of the filaments. In the simulations, coronin is localized at the top and covers the back of the actin waves. However, it is not most concentrated at the roof of the actin network, but appears to slightly lag F-actin localization with peak density relatively close to that of F-actin, as shown in Figure 12. Simulation shows that inhibition of coronin leads to actin waves with increased height and possibly alters the entire actin structure with sufficiently unbalanced branching dynamics. Alternate F-actin structures, including triggering waves and an expanding dome-like structure, may be induced by reduced coronin debranching activity, as depicted in Figure 13. These structures are similar to gelation actin waves that are caused by reduction in the effective debranching rate, observed in [18]. To study whether coronin specificity for F-actin has a role in determination of the shape of actin waves, we performed simulations on a system without coronin. It appears that coronin is not explicitly required for formation of the traveling waves as long as filament deconstruction is sufficiently compensated by spontaneous debranching. In reality, other mechanisms such as filament severing and rapid filament disintegration could account for additional filament deconstruction [36].

**Figure 14:**
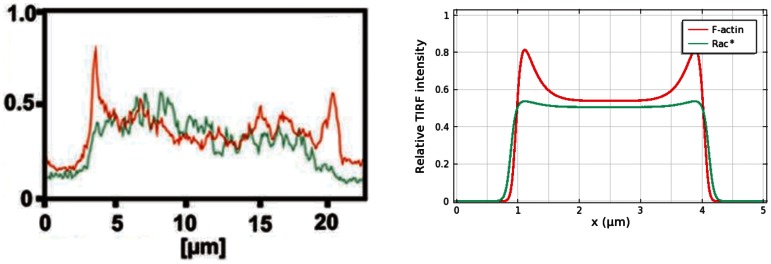
Relative localization of 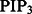 activity. TIRF images show localization of 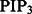 activity within the region enclosed by actin waves. *(Left)* Experimentally-observed TIRF image from [20]. *(Right)* Simulated Rac concentration is used to represent 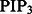 activity.

One fascinating feature of actin waves is that 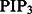 localization is enclosed by the wave fronts, with a sharp transition region near the peaks of the waves. Figure 14 displays TIRF intensity of Rac activity, which we use to represent local 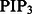 and Ras activity. The Rac activity is consistently high in the area enclosed by actin waves, in good agreement with the experimentally-observed 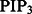 and Ras (not shown) distributions. Because Rac diffuses freely in the cytosol whereas 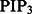 and Ras are localized at the membrane, transition regions for Rac activity extend slightly past the wave fronts while transition regions for 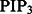 density coincide with peaks of the actin waves. It is observed that *in vivo* inhibition of PI3K by addition of LY294002 inhibits actin waves [22], and we tested the necessity of the PI3K pathway in the model by disabling activation of Rac via F-actin. The model predicts that the branching process is interrupted and actin waves cannot be formed without the PI3K activity.

### The Roles of PTEN in Stalling and Retraction of Waves and Formation of New Wave Fronts

PTEN is localized in the membrane region outside actin waves and converts 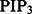 into 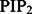. Even though its activity is not necessary for actin wave formation, cells lacking PTEN create actin waves which cannot retract. Although our model does not account for PTEN dynamics, we seek to better understand the effects of PTEN on actin wave dynamics and how retraction of the waves occurs by artificially imposing PTEN activity at specific regions on the cell-substrate surface.

**Figure 15:**
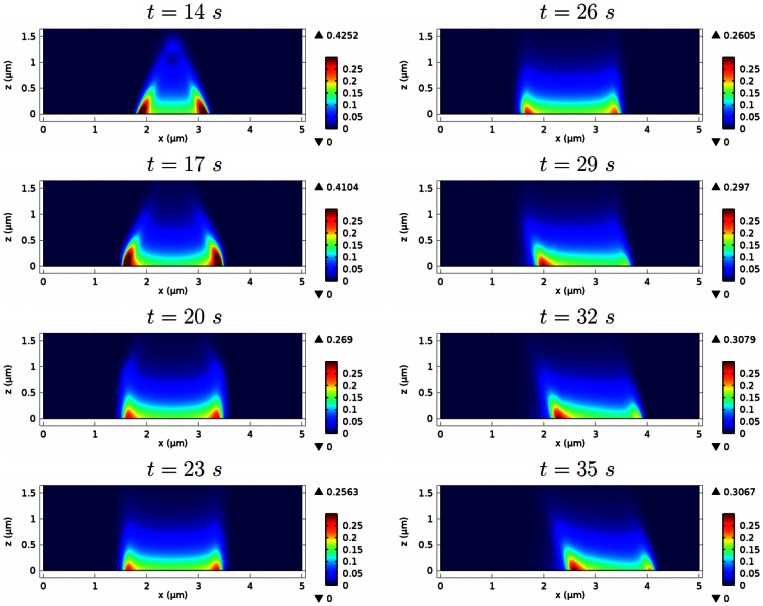
Standing and retracting actin waves. The surface density of pointed ends (in 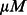) at a sequence of times. The actin waves stall between 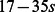, upon reaching the boundary of the PTEN region 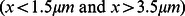. The PTEN region is stationary for 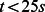 and then translates to the right at a speed of 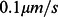, causing the wave to retract at the left and advance at the right.

**Figure 16:**
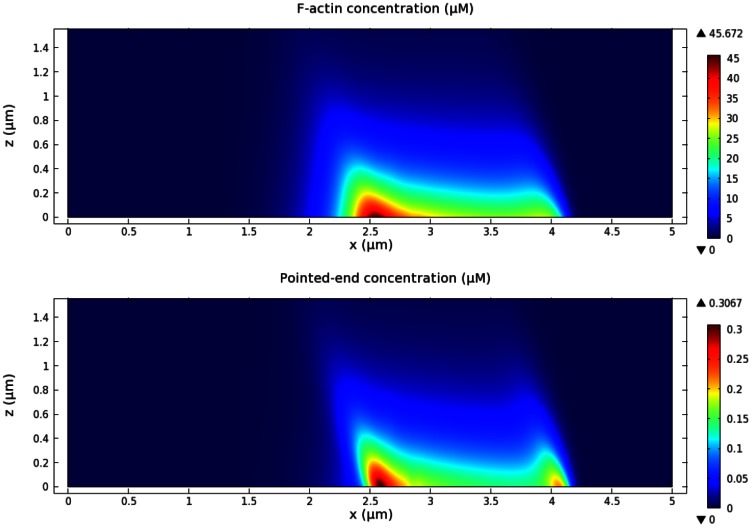
Actin structure of retracting waves. The actin density (upper) of the retracting actin waves at 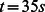 in Figure 15 corresponding to the total pointed-end density of the waves (lower).

To simulate PTEN activity, we locally disable Rac activation. When we add the PTEN activity to a fixed region, an actin wave cannot propagate through the region. Instead, its propagation is blocked near the border and the wave front becomes a standing wave. If the PTEN region pushes into the area covered by the actin wave, the wave front propagates backward as the covered area shrinks. Figures 15 displays the dynamics of a retracting wave front due to PTEN progression, and Figure 16 shows the F-actin structure of the retracting actin wave at 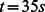. Since the peak in F-actin density is determined by the balance between available network components and the activity of activated WASP, the former of which is high outside and the latter high inside the enclosed region, receding fronts of actin waves should be present, and indeed observed, in the same fashion as the expanding fronts. A close examination of coronin localization (not shown) shows that coronin trails the wave front, in this case appearing outside the enclosed area, in good agreement with experimental observations.

**Figure 17:**
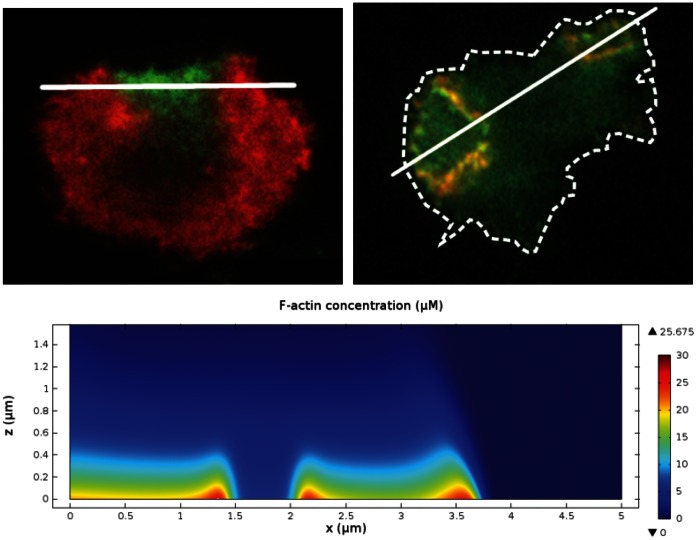
PTEN ingression and separated enclosed areas. *(Upper left)* PTEN ingression. PTEN (green) appears from the upper area of the cell-substrate contact, cutting through a portion of actin (red) wave front (adapted from [34]). *(Upper right)* Separated regions surrounded by actin waves. A TIRF image depicts F-actin (red) and Arp2/3 (green) density near the contact surface. A connected region is split into two regions after the wave front is broken at the bottom right of the cell (see [21]). New wave fronts are subsequently formed, connecting broken fronts with the existing wave front at the top left region and forming two separated regions. Dotted lines note the cell boundary. *(Lower)* A region enclosed by actin waves is divided and new wave fronts are formed by PTEN activity. New wave fronts are formed 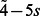 after introduction of PTEN.

Finally, we study PTEN ingression into an area covered by actin waves and separation of actin waves caused by a broken wave front. Figure 17 depicts the experimentally-observed PTEN ingression and separated actin waves, and simulations of the actin network in a vertical cross-section noted by white lines. For PTEN ingression, wave fronts along the cross-section retreat as the PTEN-covered area expands, in good agreement with the observations [34]. For separated actin waves, a broken wave front leads to formation of new wave fronts which eventually connect with existing wave fronts and separate the wave-surrounded region (see [21]). Although data on PTEN localization is not available for separation of actin waves, simulated F-actin density along the vertical cross-section when PTEN intrudes at the middle of the covered region agrees well with the experimental observations. Figure S1 displays the dynamics of new wave-front formation. Simulation suggests that introduction of the PTEN activity inhibits the positive feedback through 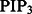 in this area, leading to eradication of the actin structure. New wave fronts are subsequently formed at the border of the region, separating the former area into two enclosed areas.

## Discussion

In this paper we presented a model of actin waves that incorporates filament dynamics and intracellular PI3K signaling, which is essential for wave formation. The network dynamics captures many fundamental processes involving F-actin, including polymerization, depolymerization, *de novo* filament nucleation, branching from mother filaments, detachment of branches, and dissociation of filaments due to depolymerization. This model allows visualization of detailed actin network structure within actin waves and is more amenable to further analysis than stochastic simulations previously studied by Carlsson [33]. Simulations of this model reveal possible mechanisms that drive propagation of actin waves. By restricting filament orientation to the vertical direction, we demonstrated that propagation of actin waves could be caused by diffusion of cytosolic proteins which regulate filament nucleation [49], although in reality the propagation speed may depend on both diffusion of the proteins and polymerization of forward-oriented filaments. Our model also highlights the role of PI3K in initiation and propagation of actin waves, as the autocatalytic cooperativity introduced by the positive feedback through 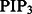 is crucial for accumulation of local F-actin density. It is suggested that a delicate balance between this positive feedback and a negative feedback that localizes cell protrusions has to be reached for efficient control of cell movement [50].

In the excitable media framework, which is a basis for existing actin-wave models, decay of the wave back is caused by activity of the slow inhibitor. Although it suggests annihilation of colliding wave fronts, it fails to explain 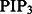 activity behind actin waves and does not allow retraction of actin waves. In contrast, our model suggests that decay of F-actin intensity at the back of actin waves may be caused by local scarcity of free cytosolic molecules such as Arp2/3 and G-actin, which are fundamental components of the actin network. The gradual decay of wave backs by depolymerization of exposed filaments leads to the observed three-dimensional structure of actin waves. This mechanism explains the experimental observation that 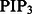 is localized in the region enclosed by wave fronts, and has the largest gradient at the wave fronts. It also predicts retraction and formation of new wave fronts when 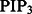 activity is locally disrupted by PTEN. Moreover, the scarcity-based decay suggests annihilation of wave fronts as they collide. Similarly, it has been reported that local scarcity of Arp2/3 or G-actin leads to retraction behind protrusion waves at the leading edge of epithelial cells [51]. In this work, filament capping, severing, and disintegration is omitted, and, to obtain adequate turnover of barbed ends to cause decay of the wave back, a relatively high depolymerization rate is needed. However, one can view this higher activity as reflective of these other processes and think of them as implicitly integrated in the depolymerization rate used in the simulations. Numerical simulations of a stochastic model (not shown here) that also includes barbed-end capping suggests that it facilitates decay of the back of actin waves.

Many molecules including Arp2/3, MyoB, CARMIL, coronin, and, in fibroblasts, integrin are associated with actin waves. Their localization is either slightly leading, slightly lagging, or coinciding with that of F-actin. Our model suggests that their localization is dictated by their roles in regulation of the actin network. Arp2/3 is an integral component of the network, responsible for branching, and thus colocalizes with F-actin. On the other hand, coronin regulates debranching and appears to decorate filaments at the top, slightly lagging the wave fronts.

Although it is unclear what gives rise to the complex dynamics of PTEN in actin waves, it plays a crucial role in the actin wave dynamics. It was observed experimentally that disruption of PTEN impairs retraction of actin waves. Our model employed a simplified description of PI3K signaling, where the activity of PTEN could not be captured, and actin waves continue expansion until they reach the cell boundary and annihilate. However, introduction of persistent PTEN activity leads to many interesting behaviors including standing waves, retracting waves, and generation of new wave fronts after intrusion of PTEN. In a future study, we plan to include more detail of the PI3K pathway to capture the behaviors caused by PTEN. In fact, complicated dynamics between 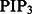 and PTEN has been observed when F-actin activity is restricted by moderate concentration of LatA [52], [53]. The full behaviors of actin waves in normal cells could be driven by this as well as the dynamics of the actin network.

There are many processes such as clathrin-coated pits and substrate-adhesion sites which may serve as precursors for actin waves. It is unclear whether actin spots are necessarily driven by these processes or whether they can survive as independent structures, and what determines if an actin spot later turns into actin waves. Our model provides a partial answer to this question, as it suggests that the precursor strength needs to exceed a threshold before it transitions into actin waves. Although we do not have information on substrate-adhesion driven precursors, the model prediction agrees well with actin spots driven by clathrin-coated pits, as some of them fail to develop into actin waves. The transition time also depends on the precursor strength, especially near the threshold. As WASP may be activated by clathrin light chains independently of 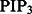 activity [41], actin spots can be observed when PI3K activity is inhibited [22]. In regard to substrate adhesion, because the F-actin nucleation and substrate-adhesion pathways potentially share common components including PI3K and F-actin [19], the activity of transmembrane receptors such as SibA, an integrin-beta homolog in *Dictyostelium*, may also be necessary for sustained activity of actin waves as well as initialization. Nevertheless, despite the influence of the precursor strength on actin-wave development, characteristics of an actin wave are independent of its initialization process. In fact, the shape and speed of actin waves are determined by rate constants and availability of actin-network constituents.

## Analysis

### The Mathematical Description of the Discrete Model

In the discrete model, state variables represent average densities of filaments and other molecular species within an interval of length 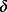, the length of an F-actin subunit (which is also the half-length of G-actin). For example, 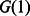 is the average density of G-actin between 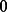 and 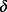, *i.e.*

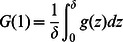
where 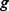 is the continuous density of G-actin and 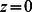 represents the membrane hypersurface. Hence, the unit of 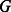 is 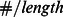. For simplicity, we first consider an infinite line 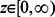 with segmentations at 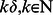.

The actin network consists of filaments of different types, distinguished by the state of their pointed ends: free (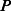), Arp2/3-bound (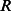), and coronin-bound (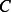). In this case, barbed ends are always at the interface 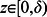 and the filaments can be defined by their length 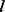, here taken at discrete locations 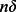, where 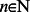, so that
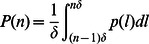
where 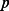 is the continuous density of free filaments. This relation provides a connection between the discrete and continuous densities. Note that the filament length coincides with distance of the filament pointed ends from the interface. In addition, we define



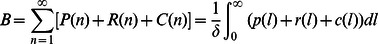
as the total amount of barbed ends per segment length. The dynamics of the filament state variables is governed by



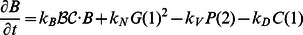


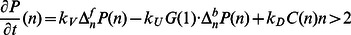


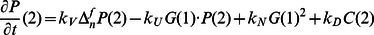


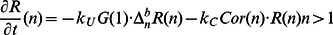


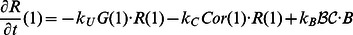


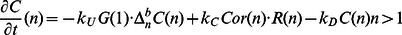


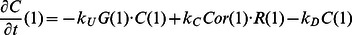
where 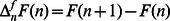, 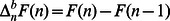 are forward and backward differences in 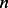. 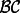 is the density of the membrane-bound branching complex, whose evolution is partly determined by intracellular signaling and will be later specified in the full model. Note that, unlike the dynamics for actin filaments *in vitro* [54], here we do not require *de novo* actin filaments to form a trimer before becoming stabilized. This is reasonable for the actin activity *in vivo*, especially in nucleation of membrane-anchored filaments which is assisted by membrane-bound proteins. The dynamics of state variables for Arp2/3 (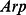), coronin (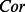), and G-actin (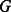) are described by



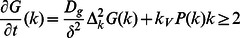


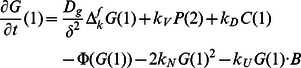


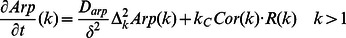


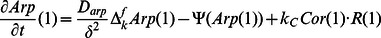


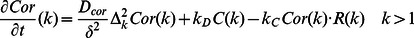


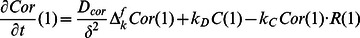
where 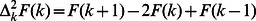 is a 2*nd*-order difference in 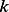. 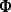 and 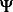 are consumption of G-actin and Arp2/3 for formation of the branching complex respectively. The description of the branching complex depends on signaling and will be defined later.

Assuming densities vanish as 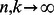, it is straightforward to check consistency of the free barbed ends
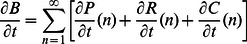
and that the total amount of actin subunits is conserved, *i.e.*



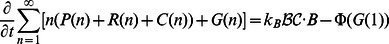



Similarly, Arp2/3 and coronin are conserved
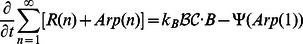


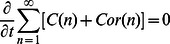



The conservation of molecules ensures consistency of the model and is essential for long-time simulations.

### The Continuum Actin Network Model as an Approximation of the Discrete Model

Because state variables in the discrete model represent average densities within intervals and the subunit length is small (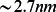) compared to the length scale of actin waves, we derive a continuum description of actin waves by deriving continuum approximations for the discrete variables. Molecular interactions within several monomer lengths from the membrane are then localized at the membrane. Any non-uniform behaviors of filaments and other molecules near the membrane may be accounted by introducing membrane species which follow dynamics implied by the discrete model. It is essential to ensure that molecular conservation is still preserved, especially for actin, which appears in both filaments and G-actin, and this is done elsewhere [55]. It can be shown that the resulting equations also conserve the total amount of Arp2/3, and coronin.

In the discrete description, 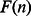 stands for an average concentration in the interval 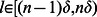. So continuous concentration 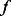 satisfies
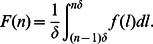



We make an additional assumption that concentrations in the continuous model are smooth so that we can use Taylor expansions. Now
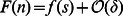


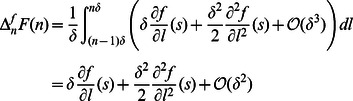


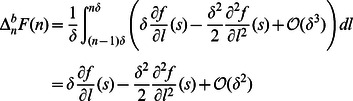


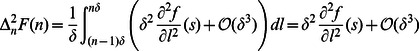
for each 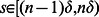 where 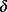 is assumed to be small compared to the characteristic length of actin wave structures. The discrete equations for filaments become



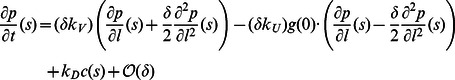


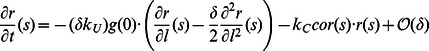


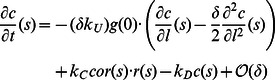
for 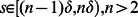. Recognizing 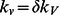 and 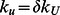 as shrinking and elongation (per subunit density) rates of actin filaments, we take the zero-order approximation of the above equations to obtain evolution equations for the actin filaments. Note that the diffusion terms are in fact first-order. They are explicitly kept to increase stability of numerical simulation, as was previously done [56]. To obtain boundary conditions for the filament densities, we model the evolution of the short filaments with length 2 or less, which have different dynamics, separately with discrete densities and use their polymerization fluxes as the boundary conditions. In particular, by taking 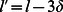, we have the following equations, for 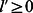,



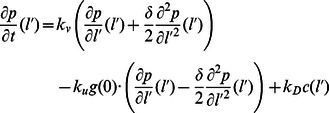


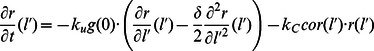


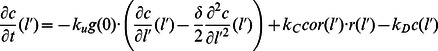



Filaments of length 3 are assumed to equilibrate quickly to their pseudo steady states
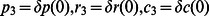
while coronin binding of 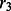 and debranching of 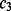 are neglected. The short filaments are defined as



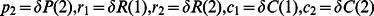
and the boundary conditions are obtained from the fluxes between filaments of length 3 and dimers. From this point, we use 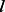 for 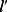 unless it is explicitly noted. Within this setting, the continuous density of uncapped filaments and the amount of short uncapped filaments are given by



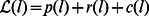


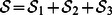
where



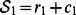


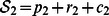


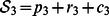
are the total amount of filaments of length 1, length 2, and length 3 respectively. Note that our pseudo steady-state assumption leads to 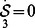 in the time scale of interest. The total amount of barbed ends is



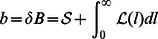



Using the zero-order approximation, we have ordinary differential equations for
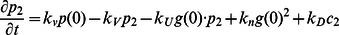


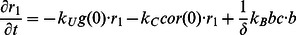


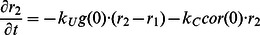


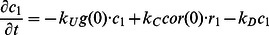


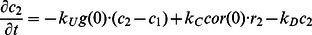
while the boundary conditions for the long filaments are



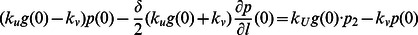


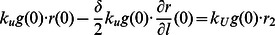


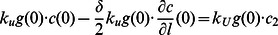
where 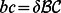 is the amount of the branching complex at the membrane and 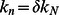.

Similarly, the zero-order approximation to the discrete equations of free molecules yields
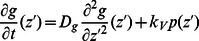


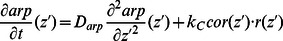


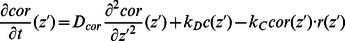
for 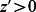, where 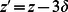. We now replace 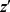 by 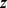 unless otherwise noted. To simplify the system, the amount of these molecules in the region 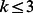 is assumed to be in the steady state and their consumption and production fluxes are used for boundary conditions



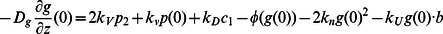


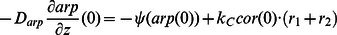


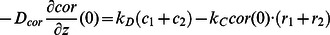
where 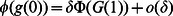 and 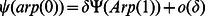. However, the total amount of actin is not preserved in this system because contributions from the second-order derivative terms (which are first-order terms in 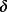) in filament equations are not accounted for in the equations for G-actin. In addition, the consumption of G-actin by polymerization of 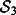 is already incorporated into the fluxes because of the pseudo steady-state assumption. To balance these contributions, correction terms for consumption and production by the second-order terms at barbed-end and pointed-end locations are introduced while the consumption by 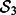 polymerization is removed to avoid double counting.



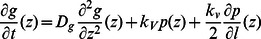


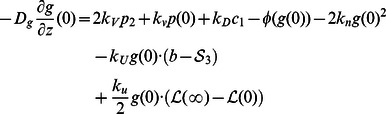
with 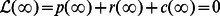. It can be shown that these correction terms lead to conservation of actin.

### The Continuum Model for the Actin Network

The transformation to a continuum description leads to a set of partial differential equations for the structure of the dendritic network and directly-related molecules as an approximation to the discrete equations. After incorporating evolution equations for other factors described in the following section, the resulting equations describe the evolution of cytosolic variables described on a rectangular domain 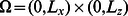 which represents a vertical cross section of a three-dimensional cell, and membrane variables defined on the lower boundary 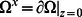. In this domain, 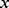 represents distance along the slice of the membrane, whereas the vertical height 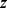 represents the direction in which filaments grow (*cf.* Figure 4).

The continuum equations for the components incorporated into the network (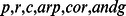) are as follows.
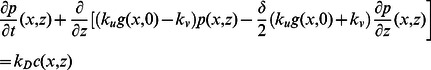


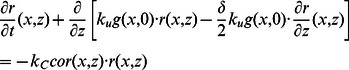


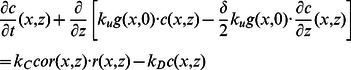


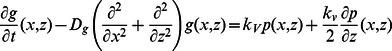


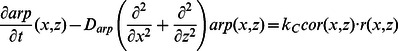


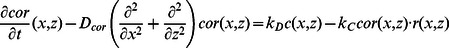



These apply in 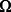, and the boundary conditions are
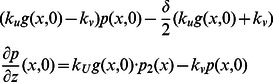


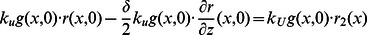


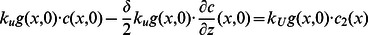


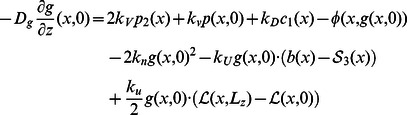


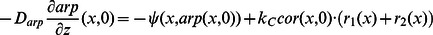


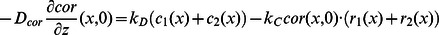
on 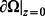 where 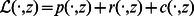 and no-flux conditions



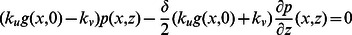


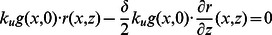


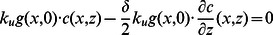


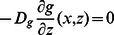


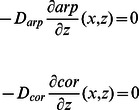
on 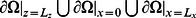. Complementary equations for short filaments are



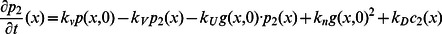


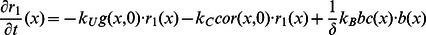


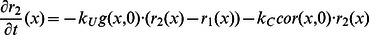


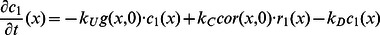


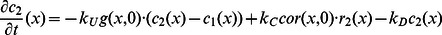
in 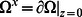 with no diffusion in 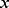 and no-flux conditions at the boundaries.

The continuum model for actin filaments is then integrated with reaction-diffusion equations for other components in the signaling network. Note that in the foregoing description polymerization is a cytosolic reaction which depends on the cytosolic concentration of free G-actin. However, in the actin wave model polymerization is localized at the boundary, *i.e.*, it involves the membrane-bound G-actin 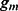 and occurs at the membrane. Therefore, the polymerization rate constant, 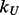, in this section is different from that used in the complete actin wave model.

### Connecting the Model Predictions with Experimental Measurements

The actin wave model describes the temporal evolution of the local concentration of filament pointed ends and other network components throughout the simulation domain. However, these quantities are not measured *in vivo*, and thus some translation of the numerical results is necessary for purposes of comparison. We first describe two imaging techniques that have been used to measure the density of molecular constituents within actin waves and then indicate how we perform the translation between the theoretically-predicted quantities and the reported measurements.

First, TIRF is used to measure the density near the lower surface of the cell. TIRF images are two dimensional and the measured intensity represents the density within 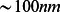 from the bottom surface. In our simulation we replicate this measurement by computing the integral of the concentration within 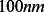 from the interface. The F-actin density is given by
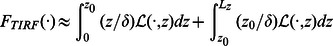
where 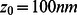 and 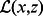 is the total pointed end density in all filament types. Note that this expression neglects the first three actin subunits of filaments. We also have



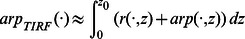


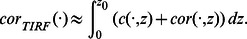



Because the 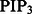 activity is integrated into the Rac-activation step, theoretically-predicted TIRF images of 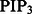 cannot be obtained directly. The best indicator for 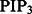 localization in our model is the level of Rac activation near the contact surface. The other imaging technique is confocal microscopy, which is used to construct z-scans which reveal the three-dimensional structure of the actin network. Molecular concentrations obtained from simulations are directly used to compare with these images. For example, the F-actin concentration at 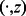 is
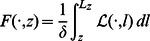



### Simulation Parameters

Protein concentrations and diffusion constants used in numerical simulations of the actin wave model are chosen within the physiological ranges. Other parameters are then obtained by matching the simulation results with experimental observations. Table 2 displays the parameter set used in the base simulation.

## Supporting Information

Figure S1
**Separation of wave covered regions and formation of new wave fronts.** Dynamics of actin waves, represented by concentration of filament pointed ends, is depicted as PTEN intrusion in a narrow region at 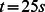 causes depletion of the F-actin and formation of new wave fronts at locations adjacent to the region.(GIF)

Movie S1 (GIF)
