# Supplementary figures and images for: A Continuum Model of Actin Waves in Dictyostelium discoideum
Source: PLoS One. 2013 May 31;8(5):e64272. doi: 10.1371/journal.pone.0064272 (PMC3669376; doi:10.1371/journal.pone.0064272)

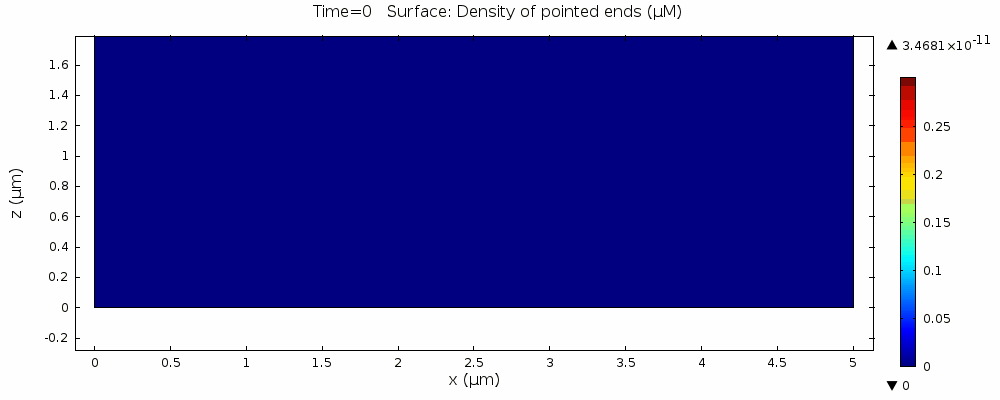

Supplement: Figure S1 — Separation of wave covered regions and formation of new wave fronts. Dynamics of actin waves, represented by concentration of filament pointed ends, is depicted as PTEN intrusion in a narrow region at causes depletion of the F-actin and formation of new wave fronts at locations adjacent to the region. (GIF) [file pone.0064272.s001.gif]
